# Effects of inhalation of low-dose nitrite or carbon monoxide on post-reperfusion mitochondrial function and tissue injury in hemorrhagic shock swine

**DOI:** 10.1186/s13054-015-0903-z

**Published:** 2015-04-22

**Authors:** Håkon Haugaa, Hernando Gómez, Donald R Maberry, Andre Holder, Olufunmilayo Ogundele, Ana Maria B Quintero, Daniel Escobar, Tor Inge Tønnessen, Hannah Airgood, Cameron Dezfulian, Elizabeth Kenny, Sruti Shiva, Brian Zuckerbraun, Michael R Pinsky

**Affiliations:** Department of Critical Care Medicine, Cardiopulmonary Research Laboratory, University of Pittsburgh, 3501 Fifth Avenue, Pittsburgh, PA 15260 USA; Department of Emergencies and Critical Care, Oslo University Hospital, Sognsvannsveien 27 0424, Oslo, Norway; Institute of Clinical Medicine, University of Oslo, Sognsvannsveien 20 0424, Oslo, Norway; Department of Surgery, University of Pittsburgh, 3380 Boulevard of the Allies 390, Pittsburgh, PA 15213 USA; Department of Critical Care Medicine, Safar Center for Resuscitation Research University of Pittsburgh, 3550 Terrace Street, Pittsburgh, PA 15261 USA; Department of Pharmacology and Chemical Biology, Vascular Medicine Institute, Center for Metabolism and Mitochondrial Medicine, University of Pittsburgh, 200 Lothrop Street, Pittsburgh, PA 15261 USA; Center for Critical Care Nephrology, University of Pittsburgh, 3550 Terrace Street, Pittsburgh, PA 15261 USA

## Abstract

**Introduction:**

Tissue reperfusion following hemorrhagic shock may paradoxically cause tissue injury and organ dysfunction by mitochondrial free radical expression. Both nitrite and carbon monoxide (CO) may protect from this reperfusion injury by limiting mitochondrial free radial production. We explored the effects of very small doses of inhaled nitrite and CO on tissue injury in a porcine model of hemorrhagic shock.

**Methods:**

Twenty pigs (mean wt. 30.6 kg, range 27.2 to 36.4 kg) had microdialysis catheters inserted in muscle, peritoneum, and liver to measure lactate, pyruvate, glucose, glycerol, and nitrite. Nineteen of the pigs were bled at a rate of 20 ml/min to a mean arterial pressure of 30 mmHg and kept between 30 and 40 mmHg for 90 minutes and then resuscitated. One pig was instrumented but not bled (sham). Hemorrhaged animals were randomized to inhale nothing (control, n = 7), 11 mg nitrite (nitrite, n = 7) or 250 ppm CO (CO, n = 5) over 30 minutes before fluid resuscitation. Mitochondrial respiratory control ratio was measured in muscle biopsies. Repeated measures from microdialysis catheters were analyzed in a random effects mixed model.

**Results:**

Neither nitrite nor CO had any effects on the measured hemodynamic variables. Following inhalation of nitrite, plasma, but not tissue, nitrite increased. Following reperfusion, plasma nitrite only increased in the control and CO groups. Thereafter, nitrite decreased only in the nitrite group. Inhalation of nitrite was associated with decreases in blood lactate, whereas both nitrite and CO were associated with decreases in glycerol release into peritoneal fluid. Following resuscitation, the muscular mitochondrial respiratory control ratio was reduced in the control group but preserved in the nitrite and CO groups.

**Conclusions:**

We conclude that small doses of nebulized sodium nitrite or inhaled CO may be associated with intestinal protection during resuscitation from severe hemorrhagic shock.

**Electronic supplementary material:**

The online version of this article (doi:10.1186/s13054-015-0903-z) contains supplementary material, which is available to authorized users.

## Introduction

The immediate restoration of blood pressure, cardiac output and oxygen-carrying capacity of patients in hemorrhagic shock is the fundamental goal of acute resuscitation. Adequate oxygen delivery to the tissues is dependent on the presence of an adequate perfusion pressure, and it is known that arterial pressure is strongly related to outcome. Patients experiencing blunt trauma who present with systolic hypotension have three times higher mortality than those who present with a normal blood pressure [1]. Furthermore, preemptive optimization of perfusion guided by oxygen delivery before surgery in high-risk patients results in a significant decrease in the rate of complications and length of stay, and once perfusion pressure is restored, optimization of cardiac output and oxygen-carrying capacity (oxygen delivery) appear beneficial [2-4]. However, reperfusion of ischemic tissue can induce reperfusion injury potentially leading to organ dysfunction and death [5].

The fundamental assumption in resuscitation physiology is that shock represents inadequate perfusion of the tissues to meet their metabolic demand and that rapid restoration of macrocirculatory perfusion pressure and blood flow will reverse this hypoperfusion, minimizing tissue injury and promoting recovery. However, it is unknown how resuscitation based on macrohemodynamic parameters impacts tissue wellness in the setting of hemorrhagic shock. Methods to quantify regional and local microcirculatory and oxygenation status have consistently shown a disconnection between macrohemodynamic parameters and tissues oxygenation and function. Mesquida *et al*. showed that tissue oxygen saturation measured in the thenar eminence by near-infrared spectroscopy had a weak correlation to mixed venous oxygen saturation (SvO_2_) [6]. More importantly, Hernandez *et al*. recently showed that dobutamine-induced increases in macrocirculatory parameters did not translate into an improved microcirculatory flow or an improved organ system function in septic patients [7]. Thus, it is unclear if manipulating the macrocirculation will proportionally benefit the microcirculation after the development of tissue ischemia.

Nitric oxide (NO) is a gaseous, short half-life molecule typically administered as continuous inhalation to ventilated neonate and adult patients. The relatively expensive and cumbersome administration has restricted the utilization of NO to critical care patients. However, systemic NO effects can also be obtained by administration of the sodium nitrite (NaNO_2_) [8]. Nitrite may be administered intravenously or nebulized as inhalation. In the body, it is reduced to NO by NO reductases such as xanthine oxidoreductase, aldehyde oxidase, deoxyhemoglobin, deoxymyoglobin, and cytochrome c oxidase [8,9].

Mitochondrial free radical production and calcium poisoning play significant roles in reperfusion injury [5]. Although the primary function of mitochondria is to convert energy derived from nutrients into adenosine triphosphate (ATP), they also produce superoxide as a byproduct of electron transport and oxygen consumption. Reactive oxygen species (ROS) and reactive nitrogen species (RNS) can be generated by superoxide dismutation and reaction with NO, respectively. In the setting of ischemia ROS/RNS production increases as electrons frequently leak from the injured electron transport change before reaching cytochrome c oxidase, potentially causing cell damage, inducing apoptosis, necrosis or both [10]. During ischemia, pharmacological preconditioning prior to resuscitation with nitrite or carbon monoxide (CO) can prevent this ‘uncontrolled’ production of free radicals by partially blocking complex I [11-14] and complex III [15] of the electron transport chain. Potentially, these small molecules may protect the cell from reperfusion injury. Based on previous studies showing that beneficial effects of nitrite and CO may be obtained with very small doses [15-17], we therefore hypothesized that exogenous administration of low-dose NaNO_2_ or CO by inhalation before resuscitation would improve electron handling by the mitochondrial electron transport chain, and would decrease tissue injury as measured in the extracellular space through microdialysis catheters [18].

## Material and methods

### Animal preparation and surgical procedure

All experiments were performed in accordance with the United States National Institutes of Health guidelines under protocols approved by the Institutional Animal Care and Use Committee of the University of Pittsburgh (protocol No. 13061614). Twenty Yorkshire Durock pigs (average weight (wt.) of 30.6 kg, range 27.2 to 36.4 kg) were acclimatized in the animal facility for at least seven days prior to study. The swine were fasted overnight but with free access to water prior to the study. Anesthesia was induced by an intramuscular injection of 0.05 mL/kg body weight (BW) of a mixture of telazole, xylazine, and ketamine, all at a concentration of 100 mg/mL. Following endotracheal intubation, the swine were ventilated with an approximate fraction of inspired oxygen (FiO_2_) of 0.6 to avoid hypoxemia and anesthesia was maintained with 1.0 to 2.5% isoflurane. A 21-gauge catheter was inserted in an ear vein and a mixture of dextrose 5% and NaCl 0.9% (Baxter, Deerfield, IL, USA) was infused at 1 mL/kg/hour thereafter.

A 7 Fr introducer was placed in the right internal jugular vein and a continuous cardiac output (CCO) pulmonary artery catheter equipped with fiber optics (Vigilance catheter, Edwards Lifesciences, Irvine, CA, USA) was floated to measure CCO and SvO_2_. A triple-lumen 18-gauge catheter for blood sampling and blood pressure monitoring using a low-volume pressure transducer (MP50, Gould Inc., Cleveland, OH, USA) was inserted in the right femoral artery, as well as an 8 Fr introducer in the right femoral vein. A urine catheter was surgically inserted in the bladder through a suprapubic incision.

Three microdialysis catheters (30 mm long, 100 kDa molecular weight cutoff membranes) (CMA 71, M Dialysis AB, Stockholm, Sweden) were inserted in the left gluteus maximus muscle, between the loops of small intestine through the suprapubic incision, and in the right lobe of the liver (right anterior medial segment V) through a 4-cm midline incision starting at the level of the xiphoid process. The microdialysis catheters in liver and muscle were placed using a splitable needle (M Dialysis AB, Stockholm, Sweden) as previously described [19].

### Hemorrhage and resuscitation protocol

**Figure 1:**
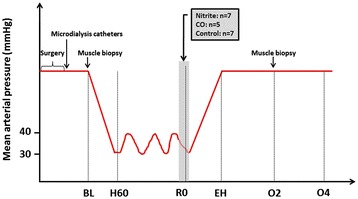
Study protocol and experimental design. In the first bleeding period (baseline (BL) to hemorrhage 60 (H60) the animals were bled at a rate of 20 mL/min. They were then kept at mean arterial pressures (MAP) between 30 and 40 mmHg for 90 minutes (H60 to start resuscitation (R0) by repeatedly hemorrhages if necessary at rates of 60 mL/min. At the end of this period the animals were administered the study drug (nitrite or CO). RO samples were obtained before administration of study drug (shadow). The animals were resuscitated with Hextend™ at a volume equal to the total bleeding amount at a rate of 60 mL/min. They were then kept at MAP equal to or above their baseline MAP using norepinephrine, and mixed venous oxygen saturation (SvO_2_) >70% using dobutamine throughout a four-hour observation period until scheduled euthanasia.

We used a modified version of a pressure-based hemorrhagic shock and resuscitation protocol previously described by us [20] (Figure 1). Briefly, after surgery swine were allowed to stabilize for 30 minutes. Using a roller pump (Masterflex™ L/S-Easy-load II, Barnant Co., Barrington, IL, USA) swine were then bled at 20 mL/min through the femoral artery until mean arterial pressure (MAP) had decreased to 30 mmHg. Repeated episodes of bleeding (at 60 ml/min) were done if the animal was able to spontaneously recover to a MAP of 40 mmHg, thus maintaining the animal between 30 and 40 mmHg for a total of 90 minutes, defined as the shock period. Resuscitation was started either when the 90-minute shock period had elapsed, or when the swine had evidence of cardiovascular collapse defined as MAP below 30 mmHg for 5 minutes or below 20 mmHg for 10 seconds. Resuscitation was done with Hextend™ (6% hetastarch with electrolytes, glucose 99 mg/dL, and lactate 28 mM (Hospira Inc., Lake Forest, IL, USA) at equal parts as shed blood, at a rate of 60 mL/min. After this initial bolus, the animals could receive crystalloids (Ringer’s lactate) and/or vasopressors to maintain baseline MAP according to a prespecified algorithm described elsewhere [20]. Animals were then observed for a 4-hour period that started at the end of Hextend™ infusion and finalized with scheduled euthanasia.

### Measurements in microdialysate and blood

The microdialysis catheters were perfused with dextran 60 and electrolytes (Plasmodex™, Meda AB, Stockholm, Sweden) at 1 μl/min^−1^ by microinjection pumps (CMA 107, M Dialysis AB, Stockholm, Sweden), as previously described by us [19]. Starting at the end of the 30-minute stabilization period samples were collected every 30 minutes and analyzed for glucose, lactate, pyruvate, and glycerol using a microdialysis analyzer (Iscus, M Dialysis AB, Stockholm, Sweden). The samples obtained after the hemorrhage period (H60) were collected prior to starting nitrite or CO in those two animal groups. After finishing the analyses the samples were frozen in liquid nitrogen and transferred to the −80°C freezer.

Simultaneously, arterial and mixed venous blood samples were taken and analyzed in a blood gas analyzer (ABL-90™, Radiometer, Copenhagen, Denmark) for pH, pCO_2_, pO_2_, SO_2_, hemoglobin, carboxyhemoglobin (COHb), methemoglobin (MetHb), glucose and lactate.

Stored plasma and microdialysate samples were subsequently analyzed as described elsewhere [21] for concentrations of nitrite by injecting plasma samples into a glass chamber containing tri-iodide, which generated NO, which was subsequently measured by ozone chemiluminescence using a Sievers 280i nitric oxide analyzer according to the instructions of the manufacturer (General Electric, Boulder, CO, USA). Plasma samples were also analyzed for aspartate aminotransferase (AST), alanine aminotransferase (ALT), and creatinine (Sigma-Aldrich assay kit, St. Louis, MO, USA).

### Mitochondrial function

Biopsies from the left rectus femoris muscle ventral of the microdialysis catheter were taken at the end of the stabilization period (baseline) and two hours after end of fluid resuscitation (Figure 1). These samples were immediately homogenized and measured after collection. Oxygen consumption in the muscle biopsies were immediately measured using a Clark-type oxygen electrode (Instech Laboratories, Plymouth Meeting, PA, USA) in the presence of succinate (for State 4 measurements) and adenosine diphosphate (ADP) (for State 3 measurement). Respiratory control ratio (RCR) was calculated as State 3/State 4.

Complex I activity: complex I activity was measured spectrophotometrically in biopsied tissue by spectrophotometrically monitoring the oxidation of nicotinamide adenine dinucleotide dehydrogenase (NADH) in the presence of coenzyme Q and the presence and absence of rotenone.

Aconitase activity: the enzymatic activity of aconitase was measured in lysed biopsies by spectrophotometrically monitoring the formation of nicotinamide adenine dinucleotide phosphate (NADPH) at 340 nm using the Bioxytech Aconitase-340 kit (Oxis Research, Foster City, CA, USA).

Carbonyl measurement: carbonyl levels were assessed in homogenized tissue spectrophotometrically (285 nm) after derivitization of the carbonyls with 2-dinitrophenylhydrazine as per the Colorimetric Carbonyl kit (Cayman Chemicals, Ann Arbor, MI, USA).

### Experimental protocol

Animals were randomized to one of the three prespecified groups at the end of first bleed: nitrite (n = 7, 30-minute nebulization of sodium nitrite at a dose of 11 mg sodium nitrite in 2.5 mL phosphate-buffered saline independent of BW); CO (n = 5, 30-minute inhalation of CO at 250 ppm independent of BW); or control (n = 7, no further intervention) (Figure 1). Microdialysis monitoring was implemented in the study protocol in the last 20 in a series of 66 pigs in total, explaining the uneven distribution of animals in each group. So there was no bias as to groups, but differences in group totals. The nitrite dose is on the low end of what was previously demonstrate to be effective in order to minimize vasodilation in the setting of shock [16]. Either nitrite or CO was started 30 minutes prior to initiation of resuscitation, 60 minutes into the ischemic shock period and continued until the end of resuscitation. One animal served as sham, by undergoing the same surgical procedure but without bleeding or administration of nitrite or CO. Additionally, we present complex I and acotinase activity and carbonyl levels from one sham animal that was included before microdialysis catheters were implemented in the protocol.

### Statistical analyses

Between-group comparisons of macrohemodynamic and systemic oxygenation parameters were performed with the Kruskal-Wallis and Mann-Whitney *U* tests. The changes in mitochondrial RCR values from baseline to the two-hour observation point were analyzed with the Wilcoxon signed-rank test and group differences were explored with the Mann- Whitney *U* test. Complex 1 and aconitase activities and levels of carbonyl were compared to controls with the Mann-Whitney *U* test. Values for AST, ALT, and creatinine at baseline and at the four-hour observation time point were analyzed with the Wilcoxon signed-rank test. Other repeated measures were analyzed in a random effects mixed model: effects of different animals, groups, BW, and amount of bleeding were included in the model aiming at achieving low information criteria. Since the data were not normally distributed the mixed model analyses were performed on log10-transformed data. Estimated fixed effects (e.f.e.) of factors and covariates are reported; values >1 represent positive effects and values <1 represent negative effects. The *P* values are two-tailed and Bonferroni-adjusted for comparison of three groups. *P* values lower than 0.05 were considered to indicate statistical significance (PASW 21.0, IBM Corp. Armonk, NY, USA).

## Results

Seven animals were randomized to the control and nitrite groups and five to the CO group. The pigs in the control group had significantly lower BW than the animals in the remaining two groups despite randomization (Table 1). All animals appeared healthy upon arrival and tolerated the surgical procedure, including insertion of microdialysis catheters, well and bleeding and resuscitation phases of the protocol were carried out without untoward complications. However, as expected based on the severity of the hemorrhagic shock protocol, three animals developed severe hypotension and died 49 (control), 51 (nitrite), and 88 (nitrite) minutes after starting resuscitation. Data from these animals were not excluded from analyses. The remaining animals were observed for a median of 253 minutes (range 230 to 260 minutes) and completed the protocol.

**Table 1:** **Body weight, surgery time, and hemorrhagic and resuscitation data in 19 pigs randomized to inhale nitrite, carbon monoxide, or no inhalation (control)**

	**Control (n = 7)**		**Nitrite (n = 7)**			**Carbon monoxide (n = 5)**		
	**Median**	**Range**	**Median**	**Range**	***P*** **value** ^**1**^	**Median**	**Range**	***P*** **value** ^**1**^
Body weight (kg)	29.5	27.2 – 31.4	33.7	30.4 – 36.4	0.004	31.1	28.0 – 35.8	0.40
Surgery time (minutes)	70	61 – 75	75	64 – 87	0.26	78	71 – 90	0.10
Shed blood volume								
mL	1000	500 – 1241	1025	761 – 1452	>0.99	711	652 – 1138	0.54
% of TBV^2^	51	27 – 70	46	38 – 63	>0.99	36	29 – 56	0.40
Duration (minutes)								
Hemorrhage 1^3^	37	28 – 55	34	26 – 42	0.77	28	23 – 33	0.06
Resuscitation	15	8 – 20	16	12 – 23	> 0.99	11	8 – 23	>0.99

^1^Nitrite and carbon monoxide groups were compared with the control group by the Mann-Whitney *U* test. The *P* values were Bonferroni-adjusted for comparison of three groups; ^2^TBV: total blood volume based on an assumed blood volume of 65 mL/kg; ^3^hemorrhage 1: time until mean arterial pressure was lower than 30 mmHg.

### Macrohemodynamic and systemic oxygenation parameters

**Figure 2:**
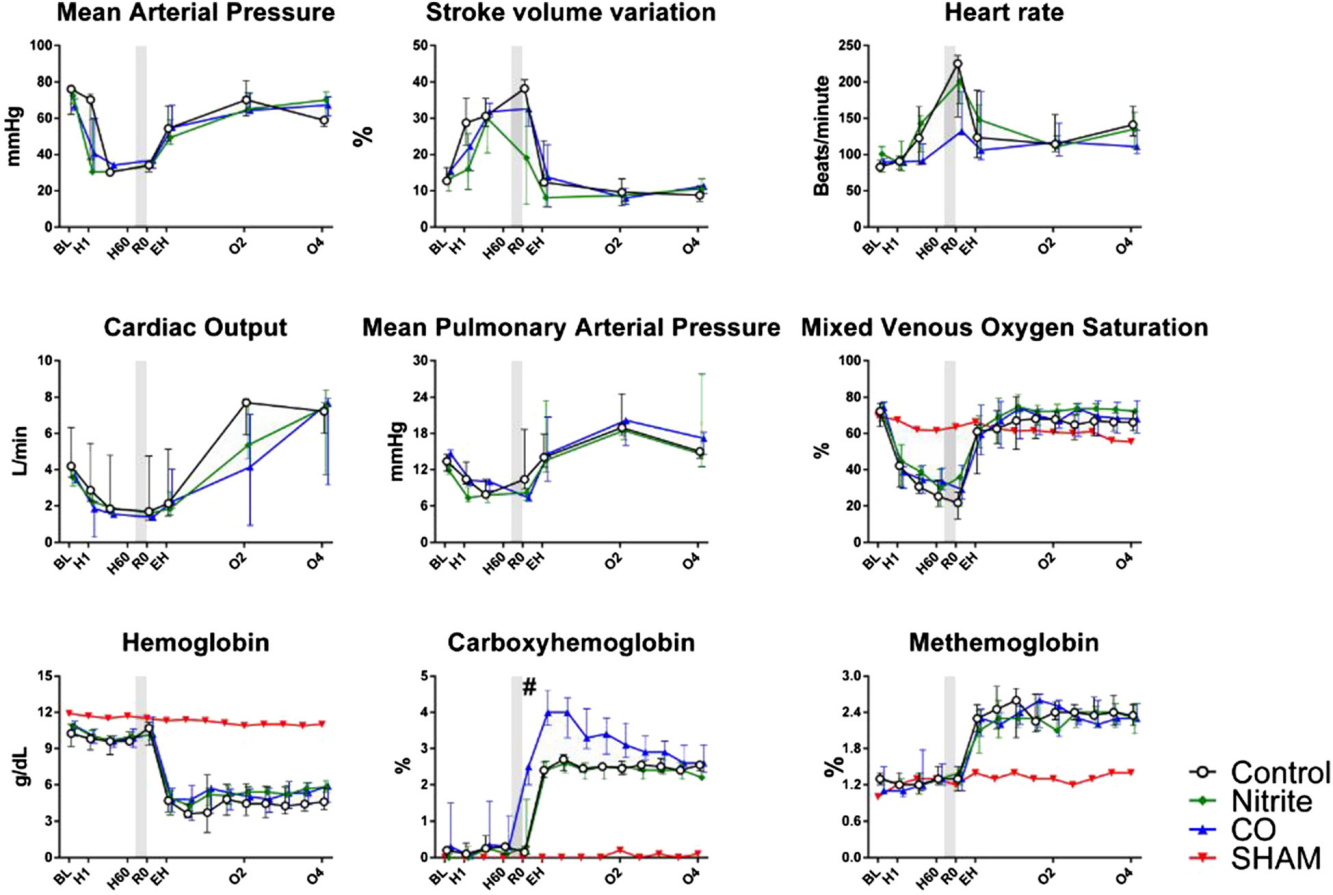
Macrohemodynamic and systemic oxygenation parameters. Hemodynamic measurements, course of mixed venous oxygen saturation (SvO_2_), hemoglobin, methemoglobin and carboxyhemoglobin in animals randomized to inhale nitrite (n = 7), carbon monoxide (CO, n = 5), or no inhalation (control, n = 7). The inhalations were administered in the shadowed period before fluid resuscitation. Values are presented as median with interquartile range. There were no group differences other than that inhalation of CO lead to an immediate increase in concentrations of CO in blood (^#^).

Hemodynamic behavior of animals followed expected trends regardless of the groups they were allocated to as summarized in Figure 2. Pharmacological preconditioning did not have any effect on MAP, stroke volume variation (SVV), heart rate (HR), CCO, or mean pulmonary artery pressure (MPAP) when compared to control animals. The MAP target of 30 mmHg (time point H0) was reached after a median of 33 minutes (range 23 to 55 minutes), with no time difference between groups (*P* = 0.20). Resuscitation was triggered by the 90-minute shock period criteria at R0 in all animals but one, in which resuscitation was started 13 minutes into the shock period for cardiovascular collapse (defined previously). All but one animal (control) required norepinephrine to keep their MAP at baseline values following resuscitation and the median maximum norepinephrine infusion rate was 0.15 μg/kg/min (range 0 to 0.4 μg/kg/min). There was no difference in the norepinephrine infusion rate between groups (*P* = 0.77). None of the animals required dobutamine.

SvO_2_ mirrored CCO and MAP changes, and hemoglobin dropped during resuscitation, as expected (Figure 2). There were no significant group differences in SvO_2_ or hemoglobin values between groups during hemorrhage or resuscitation.

### Toxicity hemoglobin parameters

We found a marked elevation of MetHb after resuscitation in all animals (e.f.e. of going from period RO to EH 1.21 (95% CI (Confidence interval) 1.18 to 1.24, *P* <0.001), independent of group (*P* = 0.84). High BW was slightly negatively correlated with MetHb (e.f.e. 0.99 (95% CI 0.98 to 1.00) (*P* = 0.05)), whereas amount of shed blood had no significant impact (*P* = 0.38) (Figure 2). Administration of CO led to an immediate increase of COHb (e.f.e. of going from H60 to RO 1.26 (95% CI 1.00 to 1.52, *P* = 0.001), independent of weight (*P* = 0.15) or amount of shed blood (*P* = 0.99). Resuscitation led to an increase of CO in all animals (e.f.e. of going from period RO to EH 1.52 (95% CI 1.41 to 1.64, *P* <0.001), and there was no difference between the nitrite and control group (*P* >0.99). The highest measured COHb in the CO group was 4.6% [22].

Microdialysis metabolic parameter trends:

1. *Nitrite* (Figure 3). During hemorrhage nitrite was unchanged in plasma, muscle, and peritoneal fluid and liver. Following inhalation of nebulized sodium nitrite prior to fluid resuscitation there was a significant increase in nitrite in plasma (e.f.e. of R0 compared to H60 to was 1.51 (95% CI 1.17 to 1.85, *P* = 0.01)). Fluid resuscitation did not lead to further increase in plasma nitrite, and until the end of the observation period, plasma nitrite decreased with an hourly e.f.e. of 0.91 (95% CI 0.86 to 0.96) (*P* = 0.003). As opposed to the nitrite group, fluid resuscitation implied an increase of plasma nitrite both in the control group and CO group; e.f.e. of EH compared to R0 in the control group was 1.16 (95% CI 1.01 to 1.32) (*P* = 0.04), and 1.22 (95% CI 1.00 to 1.43) (*P* = 0.05) in the CO group. Also different from the nitrite group, the nitrite levels did not decrease until the end of the study period neither in the control group (*P* = 0.90) nor in the CO group (*P* = 0.09). Following nitrite inhalation, concentrations of nitrite did not increase in microdialysis samples neither from muscle (*P* = 0.74) nor from peritoneal fluid (*P* = 0.44) (no samples were collected at the H60 observation time point in liver). Resuscitation did not influence nitrite concentrations in any of the microdialysis samples (*P* >0.05 for all). As opposed to the time-dependent decrease in plasma nitrite in the nitrite group after fluid resuscitation, no changes were observed in any of the microdialysis samples in the nitrite group (*P* >0.05 for all). In the CO group, the nitrite concentrations increased time-dependently in all microdialysis samples (*P* <0.05 for all). Similar changes were found in peritoneal fluid and liver in the control group (*P* <0.05 for both), whereas the increase in muscle nitrite was not significant (*P* = 0.12). The pig’s weight and amount of bleeding did not have any statistically significant influence in any of the performed statistical analyses.

**Figure 3:**
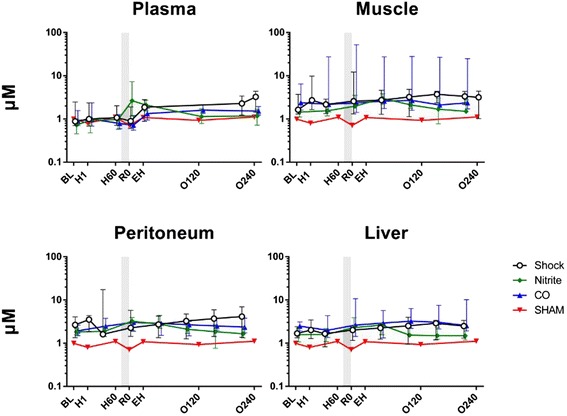
Nitrite concentrations. Nitrite values over time for nitrite (n = 7), carbon monoxide (CO, n = 5), or control (n = 7) groups. One animal was not bled (sham). The nitrite and CO inhalations timing is displayed by the shadow prior to resuscitation. Values are presented as median with interquartile range.
2. *Lactate* (Figure 4). During hemorrhage and until fluid resuscitation lactate increased at all measured sites. The steepest increase was seen in peritoneal fluid with an hourly e.f.e. of 1.26 (95% CI 1.23 to 1.29), followed by blood (1.20 (95% CI 1.17 to 1.22), liver 1.14 (95% CI 1.10 to 1.18), and muscle 1.07 (95% CI 1.05 to 1.10) (*P* <0.001 for all). There were no group differences. High amount of bleeding was positively associated with lactate in peritoneal fluid (e.f.e. of each dL hemorrhage was 1.04 (95% CI 1.02 to 1.07) (*P* = 0.02). Following resuscitation lactate continued to increase. Inhalation of nitrite had a negative effect on this continued increase in blood; the e.f.e. on lactate in arterial blood was 0.74 (95% CI 0.52 to 0.95) (*P* = 0.04). After the post-resuscitation peak lactate decreased time dependently (*P* <0.001 for all sites), and there were no group differences.

**Figure 4:**
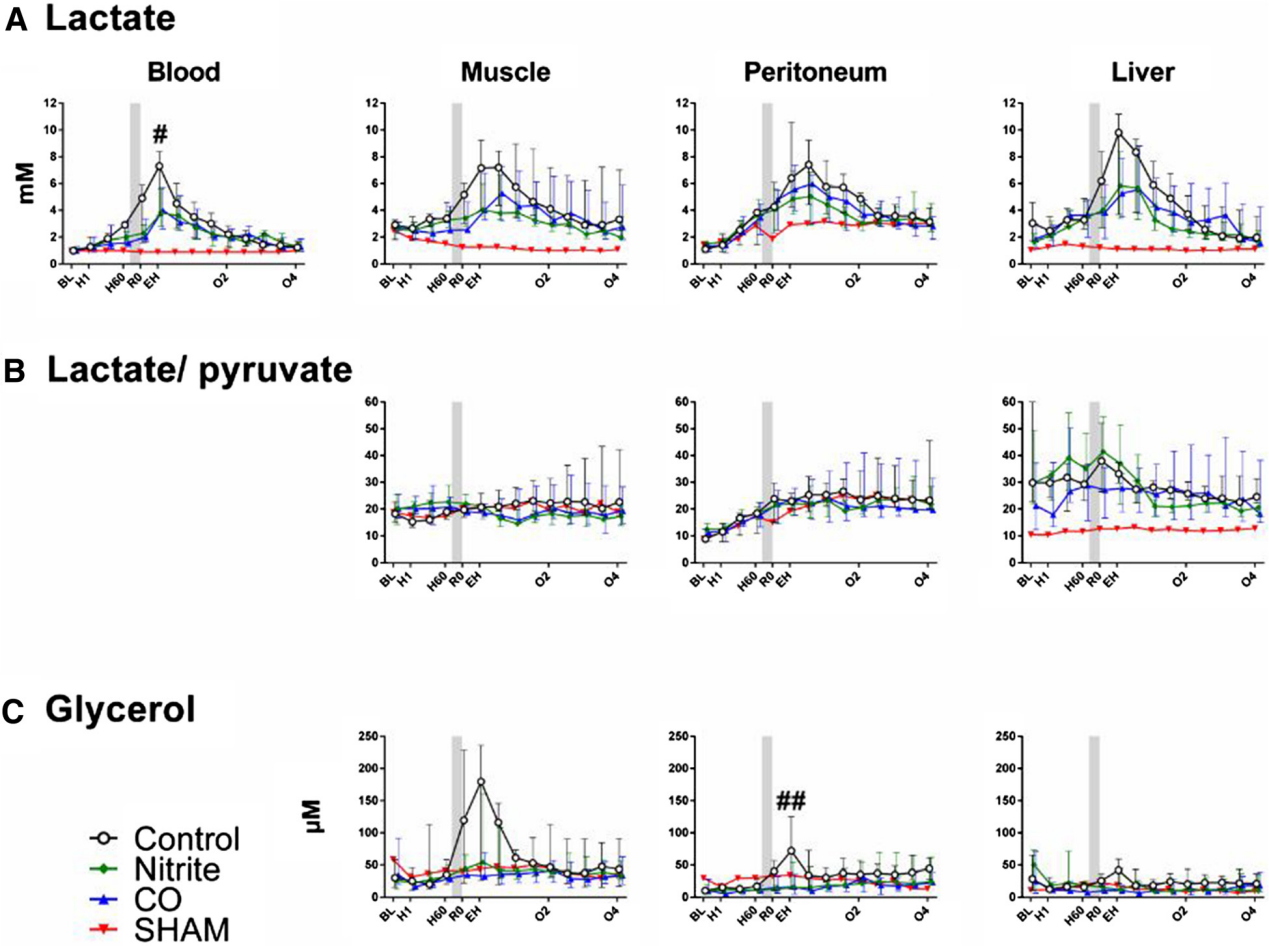
Metabolic parameters. Lactate **(A)**, lactate/pyruvate ratio **(B)**, and glycerol **(C)** values over time for nitrite (n = 7), carbon monoxide (CO, n = 5), or control (n = 7) groups. One animal was not bled (sham). The nitrite and CO inhalations timing is displayed by the shadow prior to resuscitation. Values are presented as median with interquartile range. ^#^ *P* <0.05 for the nitrite group compared to controls. ^##^ *P* <0.05 for nitrite and CO groups compared to controls.
3. *Pyruvate*. In general, the course of pyruvate concentrations throughout the experiments mirrored the lactate concentrations. As opposed to lactate, the increases during hemorrhage were not as steep as for lactate, and amount of hemorrhage had no influence on the values. There was a nonsignificant negative effect of inhaling nitrite and CO prior to fluid resuscitation.
4. *Lactate to pyruvate ratio* (*LPR*) (Figure 4). During hemorrhage, there was a time-dependent increase in LPR only in peritoneal fluid and the hourly e.f.e. was 1.14 (95% CI 1.12 to 1.16) (*P* <0.001). None of the inhaled agents had any effect on the post-reperfusion LPR values, and there were no group differences in the post-reperfusion period. Following reperfusion, no group differences were found for LPR in any of the tissues
5. *Glucose*. During hemorrhage, there was a time-dependent increase in the glucose concentrations in blood with an hourly e.f.e. of 1.04 (95% CI 1.02 to 1.06) (*P* <0.001). As opposed to blood, there were time-dependent decreases in the glucose concentrations in peritoneal fluid (e.f.e. 0.88 (95% CI 0.82 to 0.94) (*P* <0.001)), liver (e.f.e. 0.89 (95% CI 0.83 to 0.95) (*P* <0.001)), and muscle (e.f.e. 0.91 (95% CI 0.87 to 0.96) (*P* = 0.001)). Following fluid resuscitation the concentrations of glucose in tissues tended to increase, but only reaching statistically significance in peritoneal fluid with an e.f.e. of resuscitation of 1.12 (95% CI 1.00 to 1.23) (*P* = 0.04), without altering blood glucose (e.f.e. 1.00 (95% CI 0.96 to 1.04) (*P* = 0.87). Thereafter, and until the end of the observation period, glucose increased at all measured sites (*P* <0.001 for all). There was no effect of any of the inhaled agents on the post-reperfusion values.
6. *Glycerol* (Figure 4). During hemorrhage there was a time-dependent increase in the cell damage marker glycerol in muscle (hourly e.f.e. of 1.15 (95% CI 1.09 to 1.22) (*P* <0.001) and peritoneal fluid (hourly e.f.e. of 1.09 (95% CI 1.03 to 1.15) (*P* = 0.006)). Amount of bleeding was positively correlated with glycerol values (*P* <0.05 for both), and weight did not have any influence. Statistical analyses were not performed for the hemorrhage period in liver since we assume that the high initial values for glycerol were related to tissue damage caused by needle insertion. Inhalation of nitrite implied reduced release of glycerol to peritoneal fluid as compared to controls and the e.f.e. of nitrite was 0.41 (95% CI 0.10 to 0.73) (*P* = 0.002). The same tendency was observed for CO (e.f.e. 0.68 (95% CI 0.59 to 0.96) (*P* = 0.06)). Thereafter, and until the end of the study period, the values normalized and there were no group differences.

### Markers of organ damage

Plasma AST and creatinine increased significantly from samples collected at the end of the baseline period to the four-hour time point. AST increased from median 29 U/L (range 17 to 43 U/L) to 70 U/L (range 17 to 102 U/L) (*P* <0.001) and creatinine increased from median 1.0 mg/dL (range 0.2 to 1.4 mg/dL) to 1.3 mg/dL (0.2 to 1.8 mg/dL) (*P* = 0.001). As opposed to AST and creatinine, the ALT values decreased slightly from median 41 U/L (range 2 to 67 U/L) to 34 U/L (range 17 to 54 U/L). There were no group differences at any of the time points (Table 2).

**Table 2:** **Circulating markers of organ damage measured at end of baseline and at the end of the study**

	**Baseline**				**Four hours after completed resuscitation**			
	**Control (n = 7)**	**Nitrite (n = 7)**	CO* **(n = 5)**	***P*** value**	**Control (n = 6)**	**Nitrite (n = 5)**	CO* **(n = 5)**	***P*** value**
Aspartate aminotransferase (U/L)	27 (17 – 34)	30 (20 – 41)	34 (18 – 43)	0.96	68 (63 – 99)	66 (46 – 102)	73 (39 – 87)	>0.99
Alanine aminotransferase (U/L)	36 (21 – 52)	41 (28 – 67)	45 (28 – 47)	>0.99	37 (18 – 54)	26 (17 – 53)	35 (20 – 44)	>0.99
Creatinine (mg/dL)	1.0 (0.2 – 1.4)	1.0 (0.8 – 1.2)	1.2 (0.7 – 1.4)	0.83	1.4 (1.1 – 1.5)	1.2 (1.1 – 1.3)	1.3 (1.1 – 1.8)	0.37

*CO, carbon monoxide; **group differences were explored with the Kruskal-Wallis test and the *P* values were Bonferroni-adjusted for comparisons of three groups.

### Mitochondrial function

**Figure 5:**
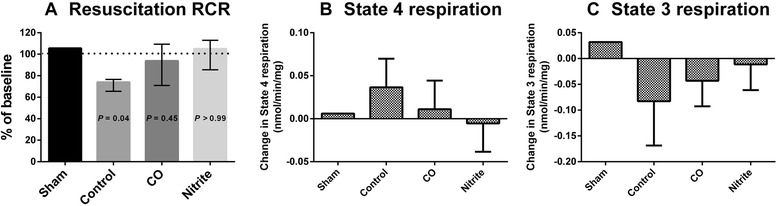
Changes in mitochondrial function after fluid resuscitation. **(A)** Respiratory control ratio for the control (n = 7), CO (n = 5), and nitrite (n = 7) groups, with one animal not bled (sham). The columns represent median values (with interquartile range as whiskers) after fluid resuscitation at the two-hour observation time point (‘O2’) as percentage of baseline values. The stapled line indicates levels at baseline. The *P* values indicate changes from baseline (Wilcoxon signed-rank test) and were Bonferroni-adjusted for comparison of the three groups. The magnitude of change in the absolute rate for State 4 **(B)** and State 3 **(C)** respiration in each group is shown.

Comparison of the RCR two hours after fluid resuscitation to the RCR in the same animal at baseline showed that RCR was significantly decreased in the control group over the course of shock and resuscitation (*P* = 0.04), but unchanged in both the nitrite (*P* = 0.45) and CO (*P* >0.99) groups (Figure 5). The magnitude of decrease in RCR observed in the control group was significantly greater than in the nitrite group (*P* = 0.05), but not compared to the CO group (*P* = 0.30). The decrease in RCR over the time course of the experiment was driven both by an increase in State 4 respiration and a decrease in State 3 in the control group, indicative of increased proton leak and decreased oxidative phosphorylation capacity respectively (Figure 5B-C).

### Antioxidant mechanisms

**Figure 6:**
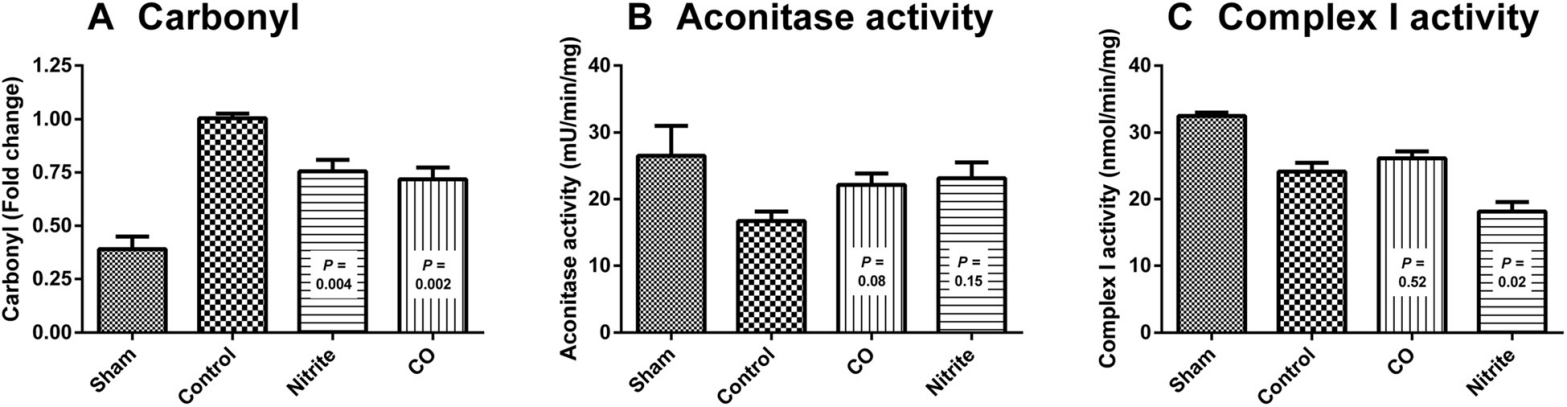
Carbonyl levels and aconitase- and complex I activity. Columns represent means +/− standard error of the mean (SEM) of **(A)** carbonyl levels, **(B)** aconitase activity and **(C)** complex I activity in sham (n = 2), control (n = 7), nitrite (n = 7) and carbon monoxide (CO) (n = 5) treated animals. The *P* values indicate comparison of treated groups to controls (Mann-Whitney *U* test) and were Bonferroni-adjusted for comparison of the three groups.

Both CO and nitrite have been shown to mediate antioxidant effects, particularly in hypoxic models [14-16]. Measurement of protein carbonyls as a marker of protein oxidation showed a significant decrease with nitrite (*P* = 0.004) and CO (*P* = 0.002) treatment compared to control animals (Figure 6A). As a second measure of oxidative stress, the activity of aconitase (an iron-sulfur enzyme particularly susceptible to oxidative inactivation) was measured. Though not significant, both nitrite (*P* = 0.15) and CO (*P* = 0.08) treatment showed a strong trend toward preserving aconitase activity (Figure 6B), consistent with the prevention of oxidative stress. Further, nitrite has been shown to inhibit complex I activity to attenuate mitochondrial ROS generation. Measurement of complex I activity showed nitrite-dependent inhibition (*P* = 0.02), but no change by CO (*P* = 0.52) (Figure 6C).

## Discussion

This study suggests that in the setting of severe hemorrhagic shock, the administration of low-dose nebulized NaNO_2_ or inhaled CO may be able to reduce the short-term ischemic and necrotic effects of reperfusion without altering macrohemodynamic and systemic oxygenation parameters although reduced concentrations of lactate were only found in blood and reduced concentrations of glycerol were only found in peritoneal fluid [19,23,24]. In accordance to this, these data also demonstrates that NaNO_2_ or CO, but not standard resuscitation alone, maintain coupling of the electron transport chain (assessed with the respiratory controlled ratio) and prevented mitochondrial dysfunction. Collectively, these findings are consistent with the hypothesis that inhaled NaNO_2_ and CO may limit reperfusion injury when used as adjuncts to standard resuscitation.

The mechanisms by which NaNO_2_ and CO exert protective effects are still unknown, although available data have consistently suggested that their main action may be at the mitochondrial level [14]. Nitrite has been shown to decrease apoptosis and cell damage in other models such as ischemia reperfusion, and across different organs, such as the heart, liver, brain and kidney [25-32]. Fast reactivation of complex I during reperfusion is recognized as a central event in ischemia/reperfusion-induced cell injury as it is related with increased production of ROS [5]. It follows that blockade of this reactivation lends a robust protective mechanism [11-13]. Recent data have suggested that nitrite may exert its protective effects through S-nitrosation of complex I of the respiratory chain. Chouchani *et al.* were able to demonstrate that nitrite selectively and reversibly S-nitrosates Cys 39 on the ND3 subunit of mitochondrial complex I [33]. This inhibition is associated with a marked reduction in mitochondria-derived ROS production, and also with reduced cell damage, necrosis and apoptosis. Our data supports these prior findings, as the respiratory control ratio, a measure of coupling of the electron transport chain, was higher in the nitrite group as compared to control.

As expected, administration of NaNO_2_ resulted in increased circulating and tissue nitrite levels. However, as shown in Figure 3, after an initial peak we found a tendency toward declining nitrite levels in our nitrite-treated group, as opposed to the control group where post-resuscitation nitrite concentrations showed an increasing tendency. In the body, NO_2_^−^ is reduced to NO by different mechanisms including enzymatic reduction by hemoglobin and myoglobin, interaction with components of the electron transport chain, and the xanthine oxidoreductase system [16,34]. Importantly, hypoxia and cell injury promote conversion of NO_2_^−^ to NO. We speculate that nitrite-induced decreases in ROS production resulted in less vascular endothelial stress reducing the normal response to this stress, which is NO production by endothelial NO synthase (eNOS). Since nitrite is the one electron oxidation product of NO and an excellent measure of eNOS activity [35], we presume the lower nitrite levels reflect lower eNOS activation. Another possibility would be that animals exposed to nitrite could have a higher rate of conversion to NO and thus, present with lower systemic levels. This does not seem to be the case as these animals had similar MAP, CCO, blood lactate, and SvO_2_ to the other groups.

Carbon monoxide blocks cytochrome c oxidase complex (or complex IV) in the mitochondrial electron transport chain, which is associated with a low level, but still increased production of ROS upstream at complex III. Acting as second messengers for CO, these ROS trigger adaptive responses and result in protection of the cell and tissues [15,36,37]. One adaptive response is the activation of the master energy regulator, adenosine monophosphate-activated protein kinase (AMPK), and subsequent stimulation of mitophagy by which dysfunctional mitochondria are self-digested in autophagosomes and their components recycled as energy substrates [38,39].

The microdialysis method is a useful way to perform repeated measurements of small molecules in the extracellular space. As such, it is a useful tool to investigate effects of preconditioning agents at the tissue or organ level [18]. Glycerol forming the backbone of the fatty acids in the cell membrane is one of these important small molecules. It is released under ischemic conditions by activation of phospholipase A2 and it is an evident marker of cell injury [19,23,40]. In the present study, the most consistent effects of pharmacologic preconditioning, as judged by lower glycerol values, were found in the peritoneal fluid, suggesting that both NaNO_2_ and CO may protect the intestine from injury caused by ischemia and subsequent reperfusion [41,42]. Since the gut mucosa is at a higher risk for ischemia/reperfusion injury than other tissues, like muscle and liver, these finding suggest a potential therapeutic role of these inhalational agents. Similar results were found in a study investigating the effects of ischemia preconditioning of heart muscle prior to coronary artery occlusion [43]. In a recent study, remote ischemic preconditioning led to reduced levels of glycerol in ischemic brain tissue [44], and similar effects of direct ischemic preconditioning have been found in liver tissue [45,46]. Furthermore, preconditioning has been shown to sustain lactate, pyruvate and the LPR in liver, muscle, free flaps and heart [46-49].

### Study limitations

Although we demonstrated effects of preconditioning agents in the extracellular space, we had only small deteriorations in organ function as measured by AST, but a 30% increase in creatinine four hours post shock. Thus, the detrimental effect of this severe hemorrhagic shock model may be dissimilar among organs and tissues. Furthermore, we did not find any group differences in AST or creatinine increases. In the setting of hemorrhagic shock, organ injury and dysfunction may not become readily apparent in the acute phase, and manifest only later following resuscitation. We reason that this was why we did not see greater evidence of organ dysfunction. Potentially, studies that include a longer window of observation may provide further insight into whether these effects on mitochondrial function will translate into protecting organ function. In addition, despite using randomization, total BW was different in the three groups, which can largely affect our results since bleeding rate was at 20 mL/min independent of BW. However, the effect of BW on the measured variables was small and furthermore, was controlled for in the statistical model. Importantly, BW had no effect on the concentrations of glycerol. A larger sham group allowing statistical comparisons would have improved the methodical strength of the study. Finally, we did not allow for any stabilization after needle and microdialysis catheter insertion in muscle and liver before starting microdialysate sampling, and thus the high baseline values in some animals most likely reflect local tissue damage inflicted by catheter insertion. Manipulation and catheter insertion in the liver shortly before obtaining baseline measures may also explain why circulating ALT was higher at baseline than at the end of the study.

## Conclusions

In conclusion, we found some evidence supporting that low-dose nebulized NaNO_2_ or inhaled CO may safely limit intestinal reperfusion injury in the setting of acute hemorrhagic shock. These data also suggest that such protective effects may be secondary to their interaction with mitochondrial respiration, although other mechanisms cannot be excluded. Both nebulized NaNO_2_ or inhaled CO hold promise as potential therapeutic adjuncts in the treatment of severe hemorrhagic shock. However, future studies will need to evaluate if these short-term effects translate into improved organ function and recovery.

## Key messages

- Low doses of nitrite or carbon monoxide may protect intestine from reperfusion injury when inhaled during hemorrhagic shock
- The effects of nitrite and carbon monoxide are probably exerted at a mitochondrial level
- Low doses of inhaled nebulized nitrite or carbon monoxide have no effects on macrohemodynamic parameters
- Microdialysis catheters allow investigating metabolic changes at organ or tissue level

## Abbreviations

ADP: adenosine diphosphate
ALT: alanine aminotransferase
AMPK: adenosine monophosphate-activated protein kinase
AST: alanine aminotransferase
ATP: adenosine triphosphate
BW: body weight
CCO: cardiac output
CI: confidence interval
CO: carbon monoxide
COHb: carboxyhemoglobin
e.f.e.: estimated fixed effect
eNOS: endothelial nitrous oxide synthase
FiO_2_: fraction of inspired oxygen
HR: heart rate
LPR: lactate to pyruvate ratio
MAP: mean arterial pressure
METHb: methemoglobin
MPAP: mean pulmonary arterial pressure
NADH: nicotinamide adenine dinucleotide dehydrogenase
NADPH: nicotinamide adenine dinucleotide phosphate
NaNO_2_: sodium nitrite
NO: nitrous oxide
RCR: respiratory control ratio
RNS: reactive nitrogen species
ROS: reactive oxygen species
S_v_O_2_: mixed venous oxygen saturation
SVV: stroke volume variation
TBV: total blood volume
wt.: weight
